# Minimally invasive perventricular device closure of ventricular septal defect in infants under transthoracic echocardiograhic guidance: feasibility and comparison with transesophageal echocardiography

**DOI:** 10.1186/1476-7120-11-8

**Published:** 2013-03-11

**Authors:** Gui-Can Zhang, Qiang Chen, Hua Cao, Liang-Wan Chen, Li-ping Yang, Dao-zhong Chen

**Affiliations:** 1 Department of Cardiovascular Surgery, Union Hospital, Fujian Medical University, Fuzhou, 350001, P. R. China

**Keywords:** Echocardiography, Congenital heart disease, Ventricular septal defects, Cardiac intervention

## Abstract

**Background:**

A hybrid approach to minimally invasive perventricular closure of VSD in infants is safe and effective, and has been performed under guidance of transesophageal echocardiography (TEE). We applied transthoracic echocardiographic (TTE) guidance to this hybrid approach, and compare results guided by TTE with those by TEE.

**Methods:**

From January 2011 to January 2012, 71 infants with VSD were enrolled to undergo a minimally invasive device closure. After evaluation of VSD by TTE, either TEE or TTE was used to guide the minimally invasive device closure. 30 patients had TEE guidance, and 41 patients had TTE. All patients were followed for 3 months after the operation.

**Results:**

The TEE group had a success rate of 93.3% (28/30) for device implantation, compared with 92.7% (38/41) in the TTE group. Two patients in the TEE group turned to surgical closure, one for involvement of the inlet area of VSD demonstrated by TEE, another for moderate aortic regurgitation after device implantation. Two patients in the TTE group also transferred to surgical closure, one for residual shunt, another for failure of the floppy wire across the defect. In addition, one patient in the TTE group experienced dropout of the occluder one day postoperatively. At 3-month follow-up, one patient had mild aortic regurgitation in the TEE group and in two patients in the TTE group. There were no episodes of cardiac block, thromboembolism, or device displacement in either group.

**Conclusions:**

TTE-guided VSD closure is feasible in infants, with results similar to those of TEE guidance, although caution is advisable.

## Introduction

Ventricular septal defect (VSD) is one of the most common forms of congenital heart disease, accounting for approximately 20% of all congenital heart disease. A large VSD may result in recurrent respiratory tract infection, pulmonary hypertension, and early heart failure in an infant. A left ventricle that is volume-overloaded due to a large defect requires clinical intervention such as surgical repair or device implantation. Percutaneous-device closure is suitable in young children, but is impractical in infants due to limitations of the delivery system. The hybrid approach such as minimally invasive perventricular closure of the VSD, introduced by Amin and coworkers in the late 1990 [1], has developed into a safe and effective method [2-6], Previously, transesophageal echocardiography (TEE) was thought to be necessary to guide perventricular device closure [7,8], and sometimes intracardiac echocardiography was used in this setting [9]. Because transthoracic echocardiography (TTE) also provides clear visualization of cardiac anatomy using subcostal views in infants [10-12], we have used TTE as a guiding tool during placement of a perventricular device [13]. Nothing is known about the potential use of TTE to guide device closure of a VSD in infants. The purpose of this study is to investigate the feasibility of TTE guidance of minimally invasive perventricular device closure of a VSD in infants, and to compare results guided by TTE with those by TEE.

## Methods

The present study was approved by the ethics committee of our university and was conducted according to the tenets of the Declaration of Helsinki. Written informed consent was obtained from the patients of their parents.

### Study patients

From January 2011 to January 2012, 71 infants (45 males and 36 females) with perimembranous VSD were enrolled in this study, and were intended to treat with perventricular device closure with domestically made VSD occluders. The patients ranged in age from 2 to 12 months (median, 7.8 months), and their weights ranged from 4 to 13 kg (median, 8 kg). The study was approved by our institutional review board, and written informed consent was obtained from the parents of all patients. All patients were routinely screened by clinical examination, chest X-rays, electrocardiography, and TTE. Pre-operative TTE was used mainly for selection of patients as candidates for implantation of a perventricular device, and exclusion other additional congenital lesions. Inclusion criteria for this study were as follows: (1) perimembranous VSD, (2) distance of over 2 mm from the rim of the VSD to nearby major cardiac structures such as the aortic, tricuspid, and pulmonary valves, (3) no aortic regurgitation, (4) maximum VSD diameter ≤10 mm. Patients were divided into the TEE group and the TTE group, according to the method used to guide device implantation. TEE was performed in 30 patients between January and July 2011, and TTE was performed in 41 patients between August 2011 and January 2012.

### Echocardiography

**Figure 1:**
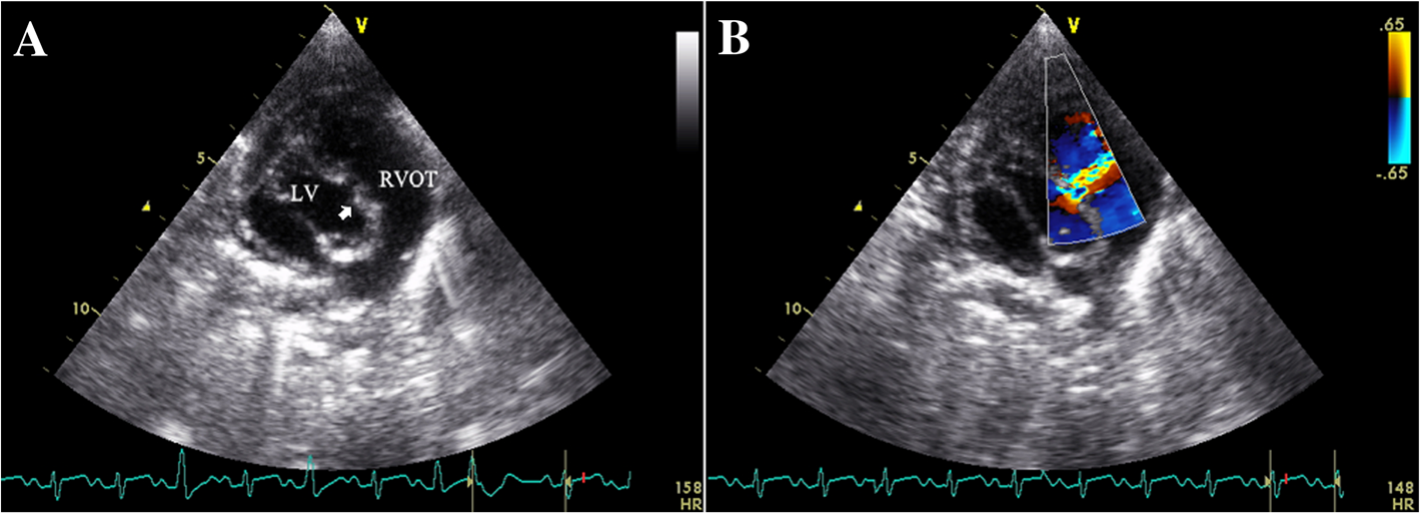
**A TTE subcostal view showing the VSD. B** The spectrum of Doppler imaging showing VSD.

All patients were examined with a complete transthoracic echocardiography using a Philip iE33 (Philip Medical System, Andover, Massachusetts) or a Vivid 7 (GE­Vingmed Ultrasound AS, Horten, Norway) imaging systems. Standard transthoracic imaging with color and Doppler interrogation from parasternal, apical, and subcostal views was performed by ultrasonic specialists to examine carefully the morphologic feature, size and rims of VSD. (Figure 1) Either an infant transesophageal probe was placed in the esophagus (TEE group), or a transthoracic probe placed below the xiphoid (TTE group) was used to guide the device implantation. TEE mid-esophagus planes were mainly used to reevaluate the VSD, and to guide the procedure in TEE-group patients.

### Device and implantation procedure

**Figure 2:**
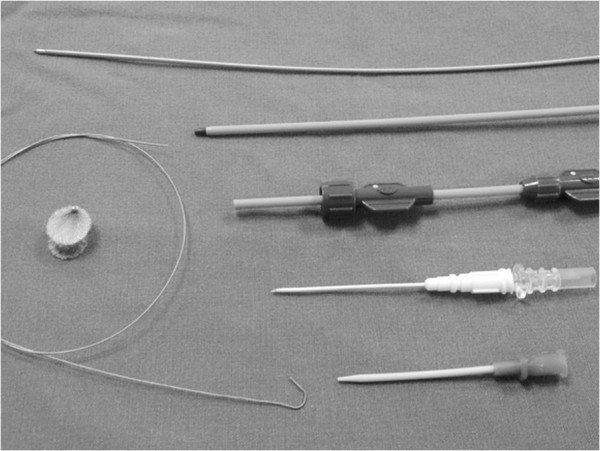
The occluder device and delivery system.

**Figure 3:**
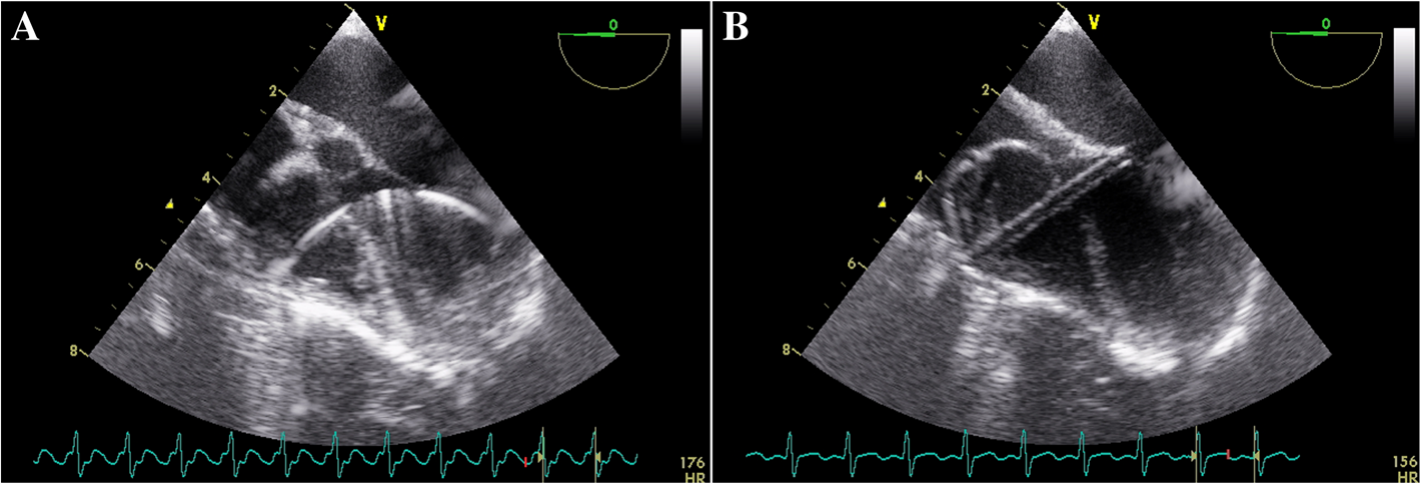
**A TEE four-chamber view showing the floppy guide wire extending through the defect. B** The delivery cable extending through the VSD into the left ventricle, with the floppy guide wire withdrawn.

**Figure 4:**
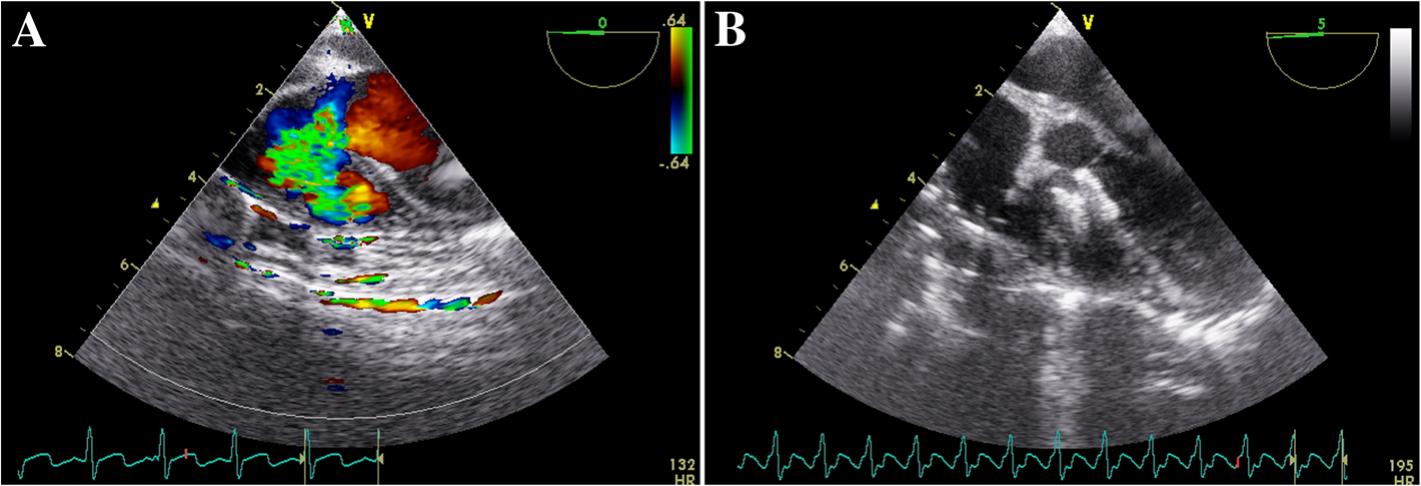
**A Color Doppler imaging of the TEE four-chamber view showing a VSD. B** Occluder was deployed with left and right disc in right position.

**Figure 5:**
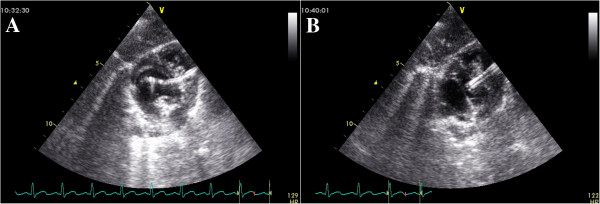
**A TTE subcostal view showing the floppy guide wire extending through the defect. B** The delivery cable extending through the VSD into the left ventricle, with the floppy guide wire withdrawn.

**Figure 6:**
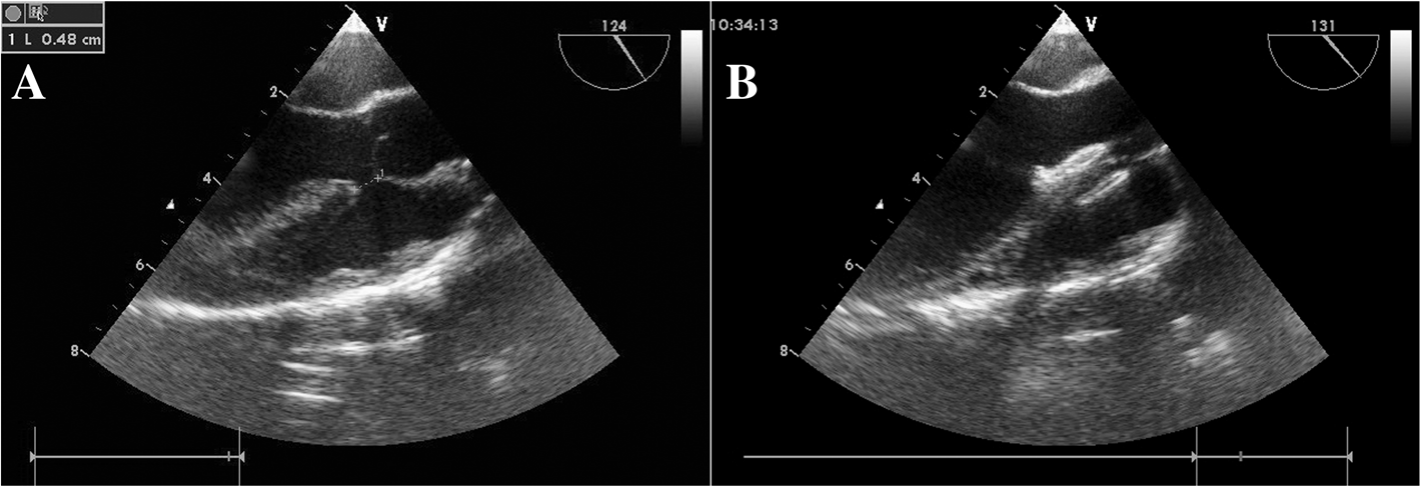
**A TEE mid-esophageal left ventricular long axis plane showing a VSD. B** An asymmetric VSD occluder was released with left and right disc along the VSD in the right position.

Two brands of domestically made VSD occluders were used in this study: the Shanghai occluders (Shanghai Shape Memory Alloy Ltd., CN, No.: 20103770832) and Shenzhen occluders (Lifetech Scientific Ltd, CN, No.: 20093770507) [14,15]. There were no much difference between these two brands of occluders, and the occluders were selected according to surgeon’s preference. Both occluders were made from an alloy of nickel and titanium, and both have two kinds of occluder: a symmetrical and an asymmetrical device (Figure 2). The diameter of the VSD was measured at diastole phase, and the occluder was selected based on this measurement plus 2 mm. After incision of the lower sternum (about 2 ~ 3 cm long), and exposure of right ventricle, heparin (1 mg/kg) was administered, and the location of puncture on the free wall of the right ventricle was determined by pinning the right ventricle toward the defect guided by echocardiography. A purse-string suture was placed, and the right ventricular free wall was punctured using a trocar. Then a floppy guide wire was inserted and advanced through the VSD into the left ventricle, and a 7Fr delivery sheath was advanced along the wire into the left ventricle under echocardiographic guidance (Figures 3, 4). The VSD occluder was loaded onto the delivery cable and introduced into the delivery sheath, and then advanced to the tip of the sheath. The left disc of the occluder was deployed when the sheath was gently pulled back until the tip was in the left ventricle, then the sheath were pulled back in the right ventricle and the left disc was closed to the ventricular septum, and the right disc of the occluder was deployed. Before release, the position of occluder disks and potential impingement of the device on adjacent cardiac structures were carefully evaluated, including assessment of residual shunt, aortic regurgitation, and atrioventricular valve function. After full evaluation, the occluder devices were unscrewed to release (Figures 5, 6), and then the sheath and delivery cable were withdrawn with the sutures tied and trimmed.

After the operation, patients were transferred and observed in an intensive care unit. A TTE examination was performed before discharge to evaluate the effects of implantation of the device. Each patient was evaluated at 3 months after the procedure.

### Statistical analysis

All continuous variables were expressed as mean ± the standard deviation or median with range as appropriate. Clinical parameters between the 2 groups were compared with the independent samples *t* test. Nominal variables were compared between the 2 groups using Fisher’s exact test. Aortic regurgitation was analyzed by the chi-square test.

## Results

The perioperative biometric data as well as the hemodynamic parameters are listed in Table 1. Occlusion was successful in 28 of 30 patients (93.3%) in the TEE group, and in 38 of 41 patients (92.7%) in the TTE group. Two patients in the TEE group turned to surgical closure, one for involvement inlet area of the VSD demonstrated by TEE, and another for moderate aortic regurgitation after device implantation. Two patients in the TTE group also transferred to surgical closure, one for failure of the floppy wire across the aneurysmal defect, and another for residual shunt (velocity great than 300 cm/s). A trivial, occluder inside residual shunt (velocity < 200 cm/s, Figure 6) through the device was observed immediately after the procedure in almost all patients in each group.

**Table 1:** The preoperative biometric data as well as the hemodynamic parameters of patients undergoing intraoperative device closure of perimembraneous VSD

	**Group TEE**	**Group TTE**	**p value**
**Number of patients**	30	41	
**Male/female**	19/11	17/24	P > 0.05
**Age (months)**	8.13 ± 0.72	7.61 ± 0.59	P > 0.05
**Body weight (kg)**	8.13 ± 0.45	7.85 ± 0.39	P > 0.05
**VSD size (mm)**	5.72 ± 0.28	5.62 ± 0.17	P > 0.05
**Occluder Size (mm)**	7.51 ± 0.31	7.51 ± 0.17	P > 0.05
**Qp/Qs**	2.45 ± 0.04	2.44 ± 0.03	P > 0.05
**operative time (minutes)**	41.1 ± 1.2	33.8 ± 0.9	P < 0.05
**aortic regurgitation (%)**	1/30	2/41	P > 0.05
**Follow-up (months)**	3.2 ± 2.1	3.5 ± 2.2	P > 0.05

Minor complications were encountered in both groups, including transient arrhythmia and occasional hypotension in the course of device implantation. One patient in the TEE group appeared to have transient hypoventilation necessitating temporary withdrawal of the TEE probe. One patient in the TTE group experienced dropout of the occluder one day postoperatively, and turned to open surgery to find a malaligned defect in the perimembranous area.

In comparison with the patients who underwent TEE-guided closure with the device, patients who underwent TTE-guided closure showed statistically similar results in mean defect size (5.7 vs. 5.6 mm), mean device size (7.5 vs. 7.5 mm), and procedure attempt success rate (92.7% vs. 93.3%) (difference not statistically significant). However, our TTE-guided group had much shorter operation time (33.8 vs. 41.1 minutes, *P* < 0.01).

The total follow-up period ranged from 1 to 6 months. The occluders remained stable, and no obstruction was observed in the left or right outflow tract. All trivial, occluder inside residual shunt that were observed resolved, except for one patient in the TTE group, who still had a mild residual shunt. Mild aortic regurgitation was found in one patient in the TEE group, and in two patients in the TTE group. There were no episodes of cardiac block, thromboembolism, or device displacement in either group.

## Discussion

Our study demonstrated that TTE may be employed for the assessment of VSD, selection of patients for device closure, and guidance of perventricular device placement in infants. Because of relatively clear definition of VSD anatomy in infants, subcostal views are practically useful in guiding the procedure of perventricular device placement.

TEE is of course the best choice for all kinds of patients in the situation of perventricular device placement because of the clear view of cardiac structures, which markedly improves assessment of device position, proximity to valve structures and residual flow. It is reported to be safe and feasible to perform transthoracic device closure of VSDs without cardiopulmonary bypass [16-19]. TTE is usually used as a tool of preparation by screening the candidates for perventricular device placement, regarding the resolution and image quality, TTE is inferior to TEE, but TTE examination in infants under general anesthesia usually have approving images, and TTE subcostal views is easy to manipulate and more welcoming by surgeons, and may be used as a guiding tool in infants with perimembranous VSD, and no patients in TTE group need to place transesophageal probe additionally because of imaging problem. Because relative deficiency of medical resource and unbalanced development in developing countries such as China, we believe that TTE-guidance may act as an alternative choice in this circumstance. When device placement is likely to be difficult, TEE-guidance is indicated, and we would also emphasize that TTE-guided VSD closure could only be carried out carefully by experienced hands.

Although morphologic variants of perimembranous VSD are common, we choose this “suitable” type of perimembranous VSD for device closure guided by TTE alone, as we know this type of VSD was more easily to apply device implantation. Here we would emphasize our inclusion criteria of VSD closure, a size of about 3 to 8 mm of perimembranous VSD may be good candidate for device closure; and an aortic rim of at least 2 mm is considered safe. In our experiences, deficiency of the aortic rim is a risk factor for unsuccessful closure and a significant predictor for residual leakage [15], A sufficient aortic rim is crucial for safe and stable positioning of the device and for prevention of impingement of the aortic valves. When aortic regurgitation does occur, we recommend using an asymmetrical device or transferring to open surgery, if new-onset mild above aortic regurgitation and residual shunting exist after the device placement, open surgery is the best choice and necessary. When pulmonary hypertension occurs with a large VSD, the ventricular septum may impinge towards left ventricle, thus affecting the parallel line of ventricular septum to the front wall of aorta, in such case may be confused as malaligned VSD. And we had encountered one event of dropout of occluder in TTE group and open surgery confirmed a malaligned defect in the perimembranous area. Careful echocardiographic evaluation is needed to exclude the malaligned VSD [20-22] because this special type of VSD is not amenable to minimally perventricular device occlusion, and must be treated with open surgery.

Compared with TEE, TTE has several disadvantages in guiding device closure of a VSD. First, because the surgical field is at the lower sternum, it is somewhat inconvenient for the ultrasonic specialist to hold the probe in a fixed position. Fortunately, by tilting the probe, it is easy to clearly show most of the main structures of the right and left inflow/outflow tracts. Secondly, the problem of contamination must be emphasized because of subcostal windows adjacent to the surgical field, therefore, we used a sterile sheath to cover the probe, and probe was placed subcostally at least 5 cm away from the surgical field. When TEE is used in infants, we have some concern about gastroesophageal injury and respiratory compression [23]. Using TTE guidance sometimes can eliminate such complications, and it is quite satisfactory to apply echo guidance with TTE in infants even less than 5 Kg.

Some technical considerations of perventricular VSD device closure should also be addressed. First, passing the guide-wire and the delivery sheath across the defect is a crucial step for successful closure. The echo reflection of the guide-wire and delivery sheath is easy to find. When passing the delivery sheath along the guide-wire, caution should be exercised in examining the tip of the delivery sheath going through the defect, at this moment right red blood may arise from the other end of the delivery sheath, this observation can also help to identify the tip of delivery sheath into the left ventricle. The surgeons and ultrasonic specialists should always be cautious not to damage cardiac structures such as the aortic or mitral valves during the procedure. Second, TEE used in the early stages of the study provided a lot of experiences, and in later stages of the study TEE probe had problem, we applied only TTE to guide VSD closure in the TTE group. Our results show that the TTE group had a reduced procedure time compared with the TEE group. Because TTE subcostal views are reliable in detection and evaluation of VSD in infants, we hope that performing the VSD closure under TTE guidance could reduce procedure time and also provide increased patient comfort.

The study is limited by the biases inherent in a retrospective registry. In addition, the decision as to which VSD closure would be most appropriate in each case was made at the discretion of the ultrasonic specialist, the surgeon, and the patient’s parents. The study was conducted in only one institution, and the sample of study was not large. A longer follow-up period is also needed to evaluate the results.

## Conclusion

Our study shows that TTE can also be used to as an alternative to guide the VSD device implantation, with caution and in experienced hands, it may be safe and feasible to perform TTE-guided VSD closure in infants.

## Competing interests

The authors declare that they have no competing interests.

## Authors’ contributions

G-CZ designed the study, collected the clinical data and performed the statistical analysis, participated in the operation and drafted the manuscript. QC, HC, L-WC and D-zC participated in the operation and revised the paper. L-pY collected the clinical data. All authors read and approved the final manuscript.
